# Excision of left ventricular papillary fibroelastoma via right minithoracotomy

**DOI:** 10.1016/j.xjtc.2022.02.021

**Published:** 2022-02-21

**Authors:** Magnus Dalén, Gustaf Sverin, Torbjörn Ivert

**Affiliations:** a Department of Cardiothoracic Surgery, Karolinska University Hospital, Stockholm, Sweden; b Department of Molecular Medicine and Surgery, Karolinska Institutet, Stockholm, Sweden

**Figure:**
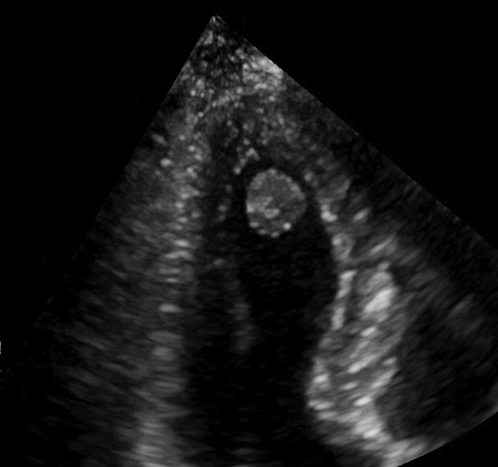
Echocardiography of a left ventricular papillary fibroelastoma.

**Central Message**

A 13-mm papillary fibroelastoma located at the apex of the left ventricle was excised via a right minithoracotomy and the mitral annulus.

Papillary fibroelastoma are benign avascular cardiac tumors usually attached with a stalk that most commonly arise at the aortic valvular endocardium with the left ventricle as the predominant nonvalvular location.^1^ The surgical options available to remove a tumor in the left ventricle are from the aorta, the mitral annulus, or via a ventriculotomy.^1^^,^^2^ We report successful excision of a papillary fibroelastoma at the apex of the left ventricle via a right minithoracotomy and the mitral annulus.

## Case Report

**Figure 1:**
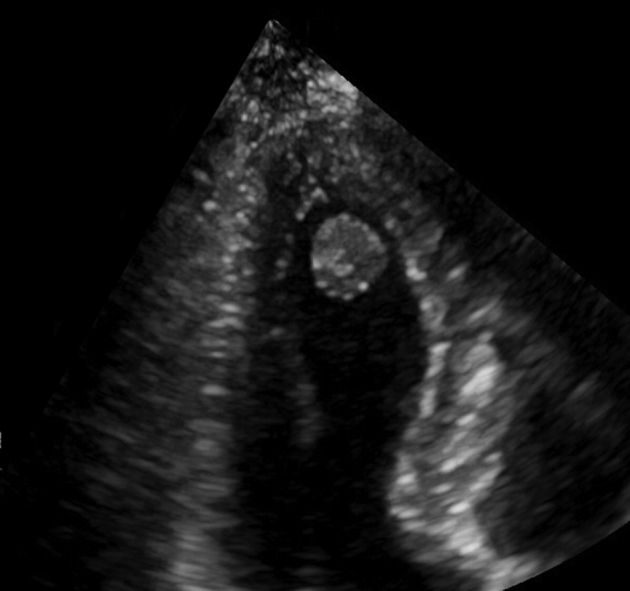
Transthoracic echocardiography of a 13-mm rounded tumor at the left ventricular apex.

**Figure 2:**
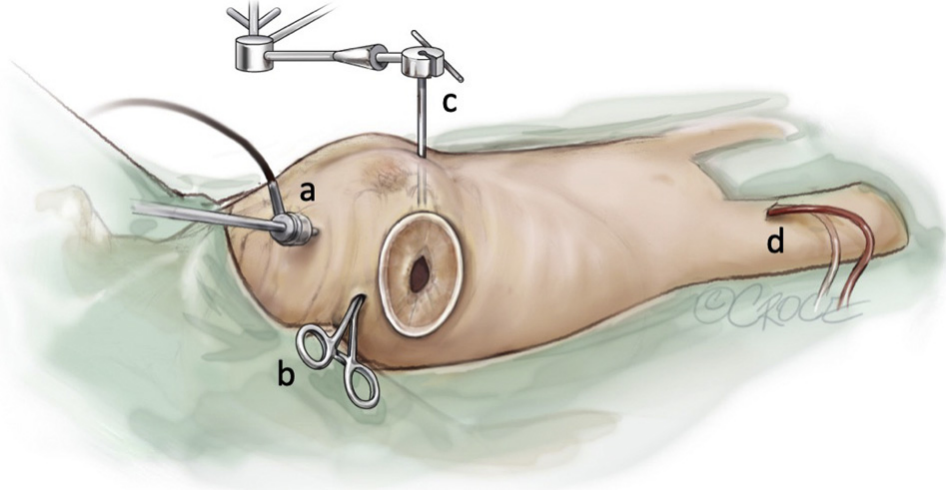
Intraoperative illustration of the surgical field and instruments used in the excision of the left ventricular tumor via a minithoracotomy. a, Camera with carbon dioxide supply. b, Transthoracic aortic crossclamp. c, Left atrial roof retractor. d, Percutaneous femoral cannulation of vein and artery (Copyright: Beth Croce, Bioperspective, Australia).

**Figure 3:**
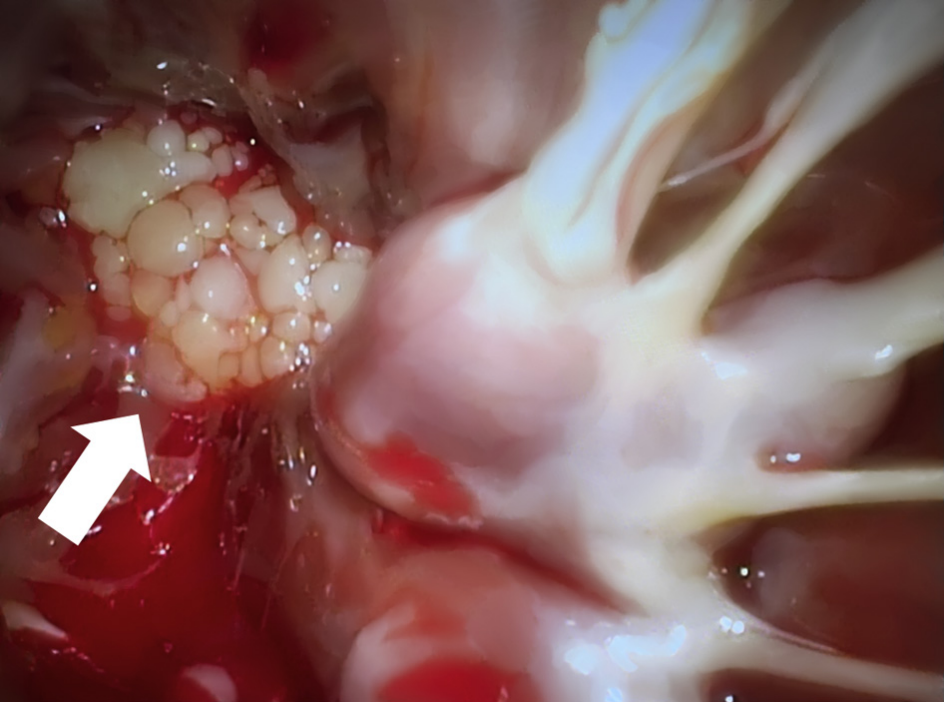
Thoracoscopic image showing the tumor located in the apex of the left ventricle (*arrow*).

A 71-year-old previously healthy woman with hypertension was admitted after a syncope. Computed tomography of the brain disclosed a small infarction. She had sinus rhythm and unremarkable laboratory tests. There were no coronary obstructions at angiography. Transthoracic echocardiography showed a 13-mm round mobile tumor at the apex of the left ventricle (Figure 1). There was no interference with valve function and normal ventricular function. A right minithoracotomy was performed with the right hemithorax slightly elevated (Figure 2). A 5-cm skin incision was made in the submammary line and the pleura entered in the fifth intercostal space. Incisions were made next to the thoracotomy, allowing access for camera with carbon dioxide supply, aortic crossclamp, left atrial drainage, and atrial roof retractor. Extracorporeal circulation was established after percutaneous femoral cannulation of vein and artery. The pericardium was incised anterior to the phrenic nerve, the ascending aorta was occluded using a transthoracic crossclamp, and cardioplegia was administered into the aortic root. The left atrium was incised anteriorly to the right pulmonary veins and the mitral valve was retracted using a self-expandable soft tissue retractor (Superflex; Fehling Instruments) allowing exposure into the left ventricle despite a small left atrium. The camera was advanced into the left ventricle. The tumor was located at the apex of the ventricle (Figure 3). By gently lifting it, the stalk was identified and cut adjacent to the myocardium. The atrial incision was sutured, the crossclamp was removed, and the patient was weaned from extracorporeal circulation. The venous cannula was removed, and the cannulation site was manually compressed for 10 minutes. The femoral artery was closed using a plug-based vascular closure device (Manta; Teleflex/Essential Medical). Distal femoral artery perfusion and correct vascular closure device location was confirmed using ultrasound. Recovery was uneventful and she was discharged from hospital after 4 days. Pathologic examination confirmed the diagnosis of papillary fibroelastoma. Low-molecular-weight heparin was given during her hospital stay but anticoagulation therapy was not started. The patient provided informed written consent for the publication of this report.

## Discussion

Cardiac papillary fibroelastoma are sometimes incidental echocardiographic findings. The correct diagnosis may be insidious as patients may present with symptoms such as systemic embolism, angina, myocardial infarction, heart failure, or sudden death.^1^^,^^2^ Therefore, when a mobile tumor in the left ventricle is diagnosed, surgery is indicated also in asymptomatic patients to prevent embolization.^1^ The papillary fibroelastoma in our patient had probably caused a small preoperative stroke. No other cause of her syncope was revealed.

Approach via right minithoracotomy is favorable in terms of visualization and invasiveness to excise pathology such as fibroelastoma, myxoma, or thrombus in the left ventricle. Access via the aorta or left ventricular wall will require a sternotomy. We used an expandable tissue retractor placed in the mitral annulus that kept the leaflets away and allowed direct complete visualization of the subvalvular structures and the tumor inside the left ventricle. The pedunculated tumor was easy to remove. There is 1 publication in Japanese and 1 report from South Korea of right minithoracotomy to excise papillary fibroelastoma in the left ventricle.^3^^,^^4^ Gastrointestinal fiberscope and thoracoscopes have been used to facilitate exposure of a papillary fibroelastoma in the left ventricle when approached via sternotomy.^1^^,^^2^ A minithoracotomy compared with sternotomy to treat atrial myxoma is associated with a lower incidence of postoperative complications and a shorter postoperative recovery period.^5^ A left ventriculotomy to resect a tumor in the left ventricle will give good exposure, but involves a risk of scar formation and arrhythmias. The advantages with a minithoracotomy are that sternotomy or ventriculotomy is avoided. Previous right thoracotomy is a relative contraindication. The possible limitation with this technique may be difficulty to visualize the exact origin of the tumor because the papillary fibroelastoma must be excised completely with its stalk at the endocardium.
